# Evaluation of a peer-delivered, transitional and post-discharge support program following psychiatric hospitalisation

**DOI:** 10.1186/s12888-017-1469-x

**Published:** 2017-08-24

**Authors:** Justin Newton Scanlan, Nicola Hancock, Anne Honey

**Affiliations:** 1 The University of Sydney, Faculty of Health Sciences, PO Box 170, Lidcombe, NSW 1825 Australia; 2 Sydney Local Health District, Mental Health Services, Sydney, Australia

**Keywords:** Discharge, Peer support, Severe mental illness, Transitional, Hospital avoidance, Recovery, Self-management

## Abstract

**Background:**

The time following discharge from psychiatric hospitalisation is a high risk period. Rates of hospital readmission are high and there is increased risk for homelessness and suicide. Transitional and post-discharge support programs have demonstrated positive results in terms of enhanced wellbeing, improved connection with community-based services and, in some cases, reductions in hospital re-admission. This paper reports on the outcomes of a peer-delivered post-discharge support program.

**Methods:**

The program involved peer workers (individuals with their own lived experience of mental illness and recovery) providing individualised practical and emotional support to individuals for six to eight weeks following discharge from an inpatient psychiatric unit. Outcomes measures included self-reported mental health recovery, personal wellness and self-reported re-admission rates. Process and satisfaction measures were also collected and semi-structured follow-up interviews were completed with consenting participants.

**Results:**

The program provided support for a total of 64 individuals, 38 of whom consented to participate in the evaluation. Participants reported improvements in terms of functional and clinical recovery and in the areas of intellectual, social and psychological wellness. Participants self-report of hospital readmissions suggested that there was a reduction in hospital bed days following engagement with the program. Themes from the follow up interviews included: *Easing the transition to the “real world”*; *Practical and individualised support; Someone to talk to; Positive qualities of the worker*, W*orkers’ lived experience was a positive thing,* and *It wasn’t long enough*.

**Conclusion:**

Overall, evaluation data suggest that the program supported positive outcomes for participants in terms of recovery, wellbeing and hospital avoidance. Participant feedback suggested that the use of support workers with their own lived experience of mental illness was a particularly powerful aspect of the program.

## Background

The time following discharge from a psychiatric hospital admission is recognised as one of the most vulnerable times for individuals with mental illness. In Australia, 14.4% of individuals are re-hospitalised within 28 days of discharge [1]. The risk of homelessness and suicide are also heightened during this period [2, 3]. Lack of connection with community-based treatment and support is considered to be a significant factor associated with all of these poor outcomes in the post-discharge period [4, 5].

In an effort to address these issues, a number of programs have been established to provide additional support to individuals during the post-discharge period. Meta-analyses [4, 6] of discharge planning and transitional support interventions have reported promising results in terms of averting re-admission and promoting engagement with community treatment. A number of different program models have been reported, however the key components of these programs have included: individualised, needs-based support; linkage with community services; and communication between inpatient and community services. A number of programs have been delivered by peer workers, that is, support workers with their own lived experience of mental illness and recovery.

One study [7] reported on a peer-delivered transitional support program in Australia. Peer workers delivered 8 to 12 h of support during the first weeks after hospital discharge. Support was focused on linking with community based treatment and support services, developing self-management strategies and other practical assistance. A total of 49 packages were delivered, with an estimated reduction of 300 hospital bed days.

Another study [5] reported results from another peer-delivered support program in the United States. Peer workers provided a “welcome basket” of daily supplies, oriented individuals to their local area and provided support to link with community agencies. Support commenced two weeks prior to discharge and continued for four weeks following discharge. Although readmission rates did not change, participants reported improvements in community functioning, life satisfaction and community integration.

Recognising the potential benefit of enhanced support around the time of discharge, a large non-government mental health service in New South Wales, Australia was funded to establish a pilot transition support program. The program, known as Hospital to Home (H2H) was rolled out in three geographical areas: two in metropolitan Sydney and one in a regional centre. The program was linked to inpatient services in each of the geographical areas. Staff from inpatient treating teams referred individuals to the program who were perceived to need additional support during the transition period. Workers in the H2H program all had their own lived experience of mental illness and recovery. Supports were tailored to the individual, but were primarily focused around providing practical and emotional support as well as linking participants with community-based supports. The program was designed to link with individuals prior to discharge and to provide approximately 6-weeks of post discharge support.

This study was led by an external, university-based research team and was established to evaluate the usefulness of the H2H program.

## Methods

The University of Sydney Human Research Ethics Committee approved the study (protocol number 2015/868). Participants who provided written, informed consent were enrolled. Data collected during the study included a range of outcome, service efficiency and satisfaction measures as well as semi-structured interviews to enable a detailed evaluation of the program.

### Outcome measures

Two primary outcome measures were used in this study. These were the Recovery Assessment Scale – Domains and Stages (RAS-DS) [8] and the Personal Wellness Wheel [9]. The RAS-DS is a self-report measure of mental health recovery that is widely used in non-government mental health services in Australia. It includes four subscales based around different domains of recovery reported in the literature: functional recovery (Doing things I value); personal recovery (Looking forward); clinical recovery (Mastering my illness); and social recovery (Connecting and belonging). Percentage scores are calculated for each domain and for “overall” recovery and higher scores represent more advanced recovery [10]. The RAS-DS has good internal and construct validity and initial evidence suggests it is sensitive to change over time [8]. The Personal Wellness Wheel is made up of eight visual analogue scales covering different aspects of wellness: Intellectual, Emotional, Physical, Social, Vocational, Environmental, Psychological and Spiritual. Participants rate their wellbeing on a scale of 0 to 10 and higher scores represent greater wellbeing [9].

In addition to these two outcome measures, estimates of pre- and post-engagement hospital bed days were calculated for participants for whom data were available. Referral documentation included number of days in hospital over the preceding year. Post-engagement hospital bed days were calculated based on self-reported mental health admission information provided by participants who completed semi-structured interviews an average of 5 months following completion of the program. To allow comparison with pre-engagement hospital bed days, this information was used to estimate number of bed days over the course of a year.

### Service efficiency measures

A number of process measures related to service efficiency were also evaluated. These included: time from referral to initial contact; duration of engagement; number of service contacts; type of service contact; and goal attainment.

### Satisfaction measures

To measure participant satisfaction, a purpose-designed 13-item questionnaire was completed at program conclusion. This questionnaire asked participants to consider how much the program had assisted them in a variety of areas including numerous aspects of community functioning, self-management and social functioning. It also included questions related to the overall usefulness of the program in terms of linking with community-based resources and avoiding hospital admissions as well as an item seeking participants views on whether having support workers who had lived experience of mental illness was helpful.

### Semi-structured interviews

Semi-structured follow-up interviews were conducted over the phone with 17 participants. All participants who provided consent and could be contacted were interviewed. Participants were asked to reflect on helpful and unhelpful aspects of their engagement with the program, as well as their relationship with their support worker. Interviews were recorded when participants consented (*n* = 11) and detailed notes taken when participants preferred (*n* = 6). Data from these interviews were coded using constant comparative analyses [11].

## Results

### Participants

Over the period that the program was operation, a total of 64 participants were engaged. Of these, 38 agreed to have their data included in the evaluation study. Demographic details of the study participants are summarised in Table 1. A large proportion of the sample was from Aboriginal or Torres Strait Islander (13.1%) or non-English speaking (34.2%) backgrounds.

**Table 1:** Demographic details for participants

Characteristic	Number (%)
Age (Mean, Standard Deviation)	45.6 years (11.2 years)
Gender	
Female	18 (47.4%)
Male	20 (52.6%)
Aboriginal and/or Torres Strait Islander background	5 (13.1%)
Non-English speaking background	13 (34.2%)
Diagnosis^a^	
Schizophrenia/Schizoaffective disorder	17 (44.7%)
Bipolar disorder	4 (10.5%)
Depression	11 (28.9%)
Anxiety	6 (15.8%)
Borderline personality disorder	3 (7.9%)
Other/Not adequately described	6 (15.8%)

^a^Diagnosis was provided in referral documentation. More than one diagnosis could be included, so totals do not add up to 100%

### Outcome measures

Pre- and post-measures for RAS-DS domains and total scores are presented in Table 2. The domains of functional recovery and clinical recovery demonstrated significant positive change over time. Pre- and post-measures for the Personal Wellness Wheel are presented in Table 3. The areas of intellectual, social and psychological wellness demonstrated significant positive change over time.

**Table 2:** RAS-DS pre- and post- scores and *t*-test results (*n* = 26)

	Pre (Mean)	Post (Mean)	*t*-test value
Total	64.6%	71.1%	*t* = 1.86, *p* = .08
Functional recovery	66.0%	76.8%	*t* = 2.76, *p* = .01*
Personal recovery	64.1%	70.3%	*t* = 1.74, *p* = .09
Clinical recovery	61.5%	69.6%	*t* = 2.06, *p* = .05*
Social recovery	67.7%	69.9%	*t* = 0.51, *p* = .61

*p < .05

**Table 3:** Personal Wellness Wheel pre- and post- scores and *t*-test results (*n* = 24)

	Pre (Mean)	Post (Mean)	*t*-test value
Intellectual	4.96	6.29	*t* = 2.16, *p* = .04*
Emotional	4.71	5.38	*t* = 1.40, *p* = .18
Physical	4.17	4.42	*t* = 0.49, *p* = .63
Social	3.83	5.00	*t* = 2.47, *p* = .02*
Vocational	3.87	4.09	*t* = 0.33, *p* = .74
Environmental	4.74	5.83	*t* = 1.76, *p* = .09
Psychological	3.92	5.38	*t* = 2.58, *p* = .02*
Spiritual	4.96	5.88	*t* = 1.55, *p* = .14

*p < .05

Post-engagement hospital beds day data were available for 18 participants. Eleven participants reported no hospital admissions since their engagement with the program. Of the remaining seven participants, two reported that they had been admitted for long-stay inpatient rehabilitation, two reported relatively lengthy admissions (> 3 weeks) and the remaining three reported short admissions (< 2 weeks). Average yearly bed days prior to engagement with the program was 68 days. Using self-reported admission information, estimated average yearly bed days following engagement with the program was 57 days. Excluding the two individuals who were subsequently admitted to inpatient rehabilitation units (which were likely planned prior to original discharge), these figures were 45 days and 26 days respectively.

### Service efficiency measures

Time from referral to first contact ranged from 0 to 76 days. Median time from referral to first contact was 2 days. Mean time from referral to first contact was 6.4 days (including four outliers) or 2.8 days (excluding outliers). The four outliers included one participant who had a long delay to discharge and three participants where service contacts did not appear to be recorded contemporaneously.

Duration of engagement with the program ranged from 13 to 209 days. Average duration was 90 days. Over this period, there were a total of 1125 service contacts related to participants, equating to an average of 30 contacts per participants. Of all contacts, 38.0% were face to face contact between the service provider and the participant. Other common types of service contacts were service coordination (232, 20.6%) and telephone contact with the participant (320, 28.4%). As would be expected given the nature of the program, service contacts were more frequent at the earlier stages of engagement and then became less frequent over time.

Information on goals was available for 30 participants. These participants set a total of 89 goals. Forty-five of these goals were reviewed with 28 (62.2%) being rated as “fully achieved” and a further 8 (17.8%) being rated as “moderate progress.” Goals were typically related to social engagement, physical health and mental health and self-care.

### Satisfaction measures

Twenty-six participants completed the end-of-program satisfaction questionnaire. The items identified as most helpful from the questionnaire included: *Having a support worker with a lived experience has helped me in my recovery* (mean 4.4 on a 5-point scale); *I feel that the support the program has given me has helped to prevent potential hospital admissions* (mean 4.3) and *The program has helped me to learn about how to access other services available in the community* (mean 4.3). The item which was least commonly affirmed by participants was *I have noticed my family relationships have improved as a result of the support I received* (mean 3.4 on a 5-point scale).

### Semi-structured interviews

Seven themes emerged from participants’ follow up interviews. These were: *Easing the transition to the “real world”*; *Practical and individualised support; Someone to talk to; Positive qualities of the worker*, W*orkers’ lived experience was a positive thing,* and *It wasn’t long enough*. Each theme is discussed below with participant quotes used to give context to each theme. All participant names are pseudonyms.

#### Easing the transition to the “real world”

For most people, H2H support commenced prior to their hospital discharge. For a few, H2H connected with them soon after discharge. All participants described how the worker assisted them to “transition to the real world… during a delicate and vulnerable time” (Charlie). Henry explained that the support of his worker provided “a safety net”.

Kelly explained that her worker helped her “To get back into the community…wasn’t easy…you don’t know what you are doing…” and the additional support made the “transition quite bearable” (Mark). Belinda said: “she welcomed me home… that’s the biggest and best thing”.

The act of picking a person up from hospital and physically driving them home or to where they would be staying in the community was a key program feature, repeatedly emphasised by participants who had connected to H2H prior to discharge. For example, Belinda said: “she just picked me up from hospital, she took me home. She had a conversation with me on the way home that made me feel good”. People also talked about the sense of safety they had when being taken home by someone they had already met through the in-reach aspect of the program: “I was respectful of the fact she took me home because I could trust her” (Belinda). In-reach not only provided this opportunity to get to know and trust the worker, it also provided some with a sense of connection to community: “when you see them at the door [of the hospital], it's basically… a connection to the outside world. I mean, I haven't been in jail but it's probably similar… where you know that someone's come from the outside and it's like an SOS type of thing.” (Susie).

One participant described her support worker taking her to numerous home visits while she was still in hospital: “I live… 45 minutes from the hospital. They would take me home a couple of times a week to do things around the house” (Laura). These home visits made her transition home easier; “like baby steps… I felt ready when I did leave” (Laura).

#### Practical support

The diverse range of practical supports provided by workers, suggests that H2H workers took an individualised approach to identifying needs of people. Some examples of practical support included: “helped me tidy my house” (Jackie); making phone calls and translating information, “whatever my personal duties, personal matters, she helped me to phone up with tremendous patience” said Joy, explaining that “my English is not very good”; “just doing the shopping, going to second-hand shops to buy clothes” (Mark); and “helping around Centrelink [Government agency responsible for disability support payments]… when my money is stopping, she’s starting it for me” (John).

Practical support for many also included helping them to linking up with appropriate services that they needed. Appropriate services for Kelly, for example, were those that would “come and pick me up” [from home], “it makes life easier” because of her mental health “it’s still hard to get out of this house” (Kelly). Not only did support workers link people with appropriate services, they facilitated them to actually access and use those services by going with them to the first visit or appointment. Susie, talking about the value of her worker driving and going with her to doctor’s appointments said: “accompanied by someone else as opposed to going by yourself… at that time I needed her to take me.” Kelly valued her worker accompanying her and explaining in simple language what doctors had said in appointments “I don’t understand what they say to me, so I get better solutions [when]… they [support worker] can tell me what they [doctor] said” (Kelly).

Participants also highlighted supports and actions of their worker that they felt were particularly ‘special’ to them and their needs and at times beyond their expectations. For James who described a long-term distress around not having enough food to eat, where “[hospital] was like a holiday… because I got three meals a day”, celebrated the fact that his worker took him to a café and “gave me a coffee and a donut” and that she “always got me home for lunch”. Talking about her worker driving her from hospital to home and to appointments after discharge, Joy said: “You feel great, because not even your family members would help you like that.” Laura talked about her isolation being the greatest of challenges and that her worker “would drop me off at my friend’s place and we’d spend a couple of hours together – she [her friend] doesn’t drive, so couldn’t come to visit me”.

#### Someone to talk to

As well as these practical actions, participants also repeatedly highlighted the value of just having someone to talk to during their transition from hospital to home. Angela said “he was very good to talk to”. Kelly said “she just talked to me. If I got upset… she’d just calm me down” and Patricia, describing her relationship with her worker said “It was like person to person. Like one person talking to another one”.

#### Positive qualities of the worker

Participants also described various attributes of their worker that they valued. While all participants described their worker in positive ways, these descriptions were diverse, including for example: “someone you can trust” (Belinda); “Some[one] that can be depended on” (Joy); “approachable… and listens” (Jackie); “Cares” (James); “Respectful – wasn’t dictating” (Mark); “Warm but professional” (Joy); “I liked her – she was nice” (Kelly), and “Wonderful, excellent … tremendous patience” (Joy). Additionally, numerous participants talked about the optimism and positivity of their worker: “Encouraging person” (Belinda), and “a bright side” (Joy) and “positive helpful” (Laura).

A common attribute of workers, highlighted by numerous participants was that they “made me [feel] safe” (Belinda). Joy described feeling “protect[ed]…I feel safe with her…I was quite frightened at that time”.

Repeatedly participants described the relationship with their support worker as being a friendship. For example James said “she was a good friend” and Belinda said “I knew straight away she was a friend”. Henry said that his worker felt “like a sister”.

#### Workers lived experience was a positive thing

While a few participants said that the fact that their worker had a personal lived experience of mental illness “didn’t make any difference”, they continued to explain that they were “really professional” (James), suggesting that they were defending against any assumption that their lived experience could be a negative. Most participants said that “this made a big difference” (Tracey) and talked about the benefits of their worker having lived experience, “it helped me to know that she had… quite a similar condition” (Belinda). They described it being helpful in developing a connection: “having gone what I’ve gone through…that’s why we connected the way we did” (Mark). A number of people also described it giving them “better empathy” (Henry). Laura, reflecting others, said that the worker’s lived experience of mental illness “meant that I knew that if I needed to talk, I would…” and “it made me feel that he did understand” (Jackie). Tracey said, contrasting it with earlier experiences with nursing staff, “I was comfortable speaking to… somebody who has the same or similar experience”.

Beyond these benefits, participants also described the hopefulness they gained from seeing the successful journey of their worker. For example, Mark said that it helped him “see the positive and the outcome of how life can be afterwards… opened my eyes a lot”. Tracey said “it was a terrific experience. It helped me to see other people with similar experiences that… can work within the community with that illness and not be judged for it”.

#### It wasn’t long enough

Most participants said that the “length that the program runs for is a bit short” (Mark) and that they needed a longer period of transition support. Charlie said “It should be longer because it could help the person become a future reality in the real world”. Joy also said “it wasn’t long enough” and went on to say that she felt like she had “kind of been cut off”.

## Discussion

This study was developed to evaluate the usefulness of a peer-delivered transition and post-discharge support program. Data were collected on outcomes (recovery, personal wellness, hospital utilisation), service efficiency and participant satisfaction. From each of these data sources, the program appears to have been valuable for participants.

Consistent with other programs reported in the literature [5–7, 12], the primary aim of H2H was to provide increased support in the post-discharge period. This increased support is designed to reduce hospital utilisation and promote participants’ recovery and wellbeing.

In this study, there was evidence of an estimated average of 11 days reduction in hospital utilisation per participant (or 19 days if data for participants admitted to long-term rehabilitation units were excluded) over the course of a year. However, these figures should be interpreted with a large degree of caution. As figures for post-engagement hospitalisations were based on self-report, these may be unreliable. Additionally, future hospital utilisation is difficult to predict and while previous utilisation is a strong predictor of future utilisation [13], it is likely that individuals’ hospital bed days will vary from year to year. Additionally, due to the high level of variance between participants’ hospital bed days in both pre- and post-engagement phases, the change in average yearly bed days did not reach statistical significance. If the 11 day reduction in hospital bed days is an accurate reflection of actual reduced utilisation, then this would represent a potential saving of $12,034 per participant, based on a cost of $1094 per day [1].

In addition to the potential savings in terms of hospital bed days, participants also reported improvements in functional and clinical recovery and several domains of wellbeing: intellectual (moving from complacent to inquisitive), social (moving from alone to connected) and psychological (moving from unadaptive to adaptive). Each of these represent positive changes experienced by participants over the time they were engaged with H2H. These results suggest that, at the conclusion of the program, participants were engaged in more meaningful activities and felt more able to manage their own mental health. In the period following hospitalisation, these are important outcomes. Supporting individuals to engaging in meaningful activities provides daily structure, opportunities to make social connections and can provide a sense of purpose, all of which have been associated with positive wellbeing [14, 15]. While improvements in clinical recovery initially seem an obvious outcome in the period following discharge, data on increased rates of suicide and re-hospitalisation following discharge suggest this is not always the case. Further, clinical recovery as defined and measured by RAS-DS refers not simply to reduced symptoms, but to reduced impact of symptoms on daily life and also to the sense of mastery over or positive management of those symptoms. Sharing of self-management strategies is one of the many benefits associated with peer-based interventions [16] and may be one of the reasons for the reported improvements in this domain of recovery.

Despite improvements in functional and clinical recovery, personal and social recovery domains of the RAS-DS did not demonstrate significant improvements over time. This is likely related to the fact that improvements in these areas may take longer to emerge. There are, however, substantial indicators that positive changes had occurred in these areas. Firstly, average scores in each of these domains were higher at the conclusion of the program than at commencement. Secondly, positive relationships with support workers with lived experience of mental illness and recovery were highlighted by interview participants as helping to ignite their hope for the future and provided valuable social contact during the vulnerable post-discharge period. This is also reflected in significant improvements in the domains of psychological and social wellbeing in the Personal Wellness Wheel.

Lack of social connectedness is a commonly identified issue for individuals living with mental illness [17] and is challenging to overcome [18]. While positive relationships with H2H workers were helpful, it may be useful to increase focus on family and other natural and thus sustainable relationships. A Tomita, EP Lukens and DB Herman [2] reported positive outcomes associated with family-focused interventions in a transitional support program; improved satisfaction with family relationships was associated with a modest reduction in psychiatric re-hospitalisation rates. Additionally, having a variety of friends and being engaged in relationships where individuals provide support to others have been reported as important indicators of more advanced recovery [19, 20].

The value of the lived experience of support workers was highlighted by participants in both the satisfaction questionnaire and interviews. Numerous advantages of peer-support interventions have been highlighted in previous research [16, 21, 22] and are reflected in the increased employment of peer workers by government and non-government mental health services in Australia and internationally. Similar to results from other studies of peer-delivered transition support programs [5, 7], participants in this study highlighted that support workers were understanding, demonstrated empathy and had more credibility due to their own lived experience of mental illness. Additionally, participants identified that support workers were positive role models, supporting an increased sense of hopefulness for the future.

Finally, supports provided through the H2H program were valued by participants. Information from satisfaction questionnaires and interviews highlighted those features most valued by participants. Participants valued support to connect with community-based resources, emotional supports offered and practical supports provided. Notably, participants highlighted the value of assistance provided in terms of transportation, particularly being transported from hospital to home at the point of discharge and when completing necessary activities in the period following discharge. This is an important finding, as these practical supports are often viewed as unnecessary and a poor utilisation of resources. Additionally, these benefits associated with these practical supports may not be captured by typical service evaluation measures or by clinical or recovery-oriented outcome measures. Finally, the prompt actioning of referrals, frequent face-to-face contacts and positive results in terms of personal goal attainment further demonstrate the responsiveness and usefulness of this program.

### Limitations

There are a number of limitations associated with this study that should be highlighted. Firstly, the relatively small number of participants and absence of a specific control group limits the generalizability of these results to other settings. While positive changes were reported by participants, it is not possible to definitively conclude that these changes were a direct result of their involvement with H2H. Potentially, these changes could have been related to other interventions received, or due to natural improvements that occur over time. Secondly, hospitalisation data for the post-engagement period were based on self-report and only available for a sub-sample of participants. This is a serious limitation and means that it is not possible to make definitive conclusions about the effectiveness of the program in reducing hospital admissions. It is possible that the self-report data of may be inaccurate as participants all have serious mental illnesses and may experience cognitive difficulties.

## Conclusions

Overall, evaluation data suggest that the H2H program has resulted in positive outcomes for participants. Positive outcomes were achieved in recovery and wellness and participant feedback suggests that the use of support workers with their own lived experience of mental illness was a particularly powerful aspect of the program. Additionally, the measures of service efficiency suggest that the program provided timely and efficient services to participants. Finally, although it is not possible to draw definitive conclusions, there is some preliminary data that suggest the program may have supported reductions in hospital utilisation.

For future research in this area, it would be valuable to complete studies that include a control condition and complete more rigorous analyses of post-engagement service utilisation (both community-based and hospital-based resources). Additionally, specific impact analyses including cost-benefit analyses would be useful. This would provide a more detailed understanding of the overall contribution of these programs.

## Abbreviations

H2H: Hospital to Home
RAS-DS: Recovery Assessment Scale – Domains and Stages

## References

[1] Key performance indicators for Australian public mental health services [https://mhsa.aihw.gov.au/indicators/nkpi/].

[2] Tomita A, Lukens EP, Herman DB (2014). Mediation analysis of critical time intervention for persons living with serious mental illnesses: assessing the role of family relations in reducing psychiatric rehospitalization. Psychiatric Rehabilitation Journal.

[3] Olfson M, Wall M, Wang S (2016). Short-term suicide risk after psychiatric hospital discharge. JAMA Psychiatry.

[4] Steffen S, Kösters M, Becker T, Puschner B (2009). Discharge planning in mental health care: a systematic review of the recent literature. Acta Psychiatr Scand.

[5] Kidd SA, Virdee G, Mihalakakos G, McKinney C, Feingold L, Collins A, Davidson L, Weingarten R, Maples N, Velligan D (2016). The welcome basket revisited: testing the feasibility of a brief peer support intervention to facilitate transition from hospital to community. Psychiatric Rehabilitation Journal.

[6] Vigod SN, Kurdyak PA, Dennis CL, Leszcz T, Taylor VH, Blumberger DM, Seitz DP (2013). Transitional interventions to reduce early psychiatric readmissions in adults: systematic review. Br J Psychiatry.

[7] Lawn S, Smith A, Hunter K (2008). Mental health peer support for hospital avoidance and early discharge: an Australian example of consumer driven and operated service. J Ment Health.

[8] Hancock N, Scanlan JN, Honey A, Bundy AC, O’Shea K (2015). Recovery assessment scale – domains and stages (RAS-DS): its feasibility and outcome measurement capacity. Aust N Z J Psychiatry.

[9] Gill K (2012). New moves: targeting physical and mental well-being in people with mental illness. Health Issues.

[10] Hancock N, Scanlan JN, Bundy AC, Honey A (2016). Recovery assessment scale - domains and stages (RAS-DS) manual. Version 2.

[11] Charmaz K (2014). Constructing grounded theory.

[12] Reynolds W, Lauder W, Sharkey S, Maciver S, Veitch T, Cameron D (2004). The effects of a transitional discharge model for psychiatric patients. J Psychiatr Ment Health Nurs.

[13] Kisley S, Preston N, Xiao J, Lawrence D, Louise S, Crowe E, Segal S (2013). An eleven-year evaluation of the effect of community treatment orders on changes in mental health service use. J Psychiatr Res.

[14] Iredale C, Fornells-Ambrojo M, Jolley S (2016). Psychological interventions for housebound people with psychosis: service user and therapist perspectives in south East London. J Ment Health.

[15] Leufstadius C, Erlandsson LK, Eklund M (2006). Time use and daily activities in people with persistent mental illness. Occup Ther Int.

[16] Repper J, Carter T (2011). A review of the literature on peer support in mental health services. J Ment Health.

[17] Cleary M, Freeman A, Hunt GE, Walter G (2006). Patient and carer perceptions of need and associations with care-giving burden in an integrated adult mental health service. Soc Psychiatry Psychiatr Epidemiol.

[18] Hancock N, Scanlan JN, Gillespie J, Smith-Merry J, Chen I. Partners in Recovery program evaluation: changes in unmet needs and recovery. Aust Health Rev. 2017; doi:10.1071/AH17004. 10.1071/AH17004 28693718

[19] Hancock N, Bundy A, Honey A, Helich S, Tamsett S (2013). Measuring the later stages of the recovery journey: insights gained from clubhouse members. Community Ment Health J.

[20] Hancock N, Honey A, Bundy AC (2015). Sources of meaning derived from occupational engagement for people recovering from mental illness. Br J Occup Ther.

[21] Miyamoto Y, Sono T (2012). Lessons from peer support among individuals with mental health difficulties: a review of the literature. Clin Pract Epidemiol Ment Health.

[22] Trachtenberg M, Parsonage M, Shepherd G, Boardman J (2013). Peer support in mental health care: is it good value for money?.

